# Supplementary material for: Prognostic Significance of the Relative Load of KPC-Producing Klebsiella pneumoniae within the Intestinal Microbiota in a Prospective Cohort of Colonized Patients
Source: Microbiol Spectr. 2022 Jun 29;10(4):e02728-21. doi: 10.1128/spectrum.02728-21 (PMC9431423; doi:10.1128/spectrum.02728-21)

Supplementary Material

Index

Index 1

Supplementary Methods 3

Culture-based and qPCR-based quantification of the relative load of KPC-KP in rectal swabs 3

Supplementary Tables 5

Supplementary Table S1. Checklist of items according to STROBE document. 5

Supplementary Table S2. Linearity of qPCR assays. 8

Supplementary Table S3. Antimicrobial susceptibility profiles of 80 KPC-producing *Klebsiella pneumoniae* isolates. 9

Supplementary Table S4. Clinical characteristics and outcomes for 33 infection episodes. . 10

Supplementary Table S5. Univariable competing risk Fine-Gray regression model for the first KPC-KP infection episode *versus* death from other causes (considered as competing risk) within 90 and 30 days of follow-up in the global cohort. 12

Supplementary Table S6. Univariable and multivariable Cox regression analyses of risk factors for 90-day mortality in the global cohort of 80 patients. 13

Supplementary Table S7. Univariable and multivariable Cox regression analyses of risk factors for 30-day mortality in the global cohort of 80 patients. 16

Supplementary Figures 18

Supplementary Figure S1. Graphical summary of rectal swab processing in this study (KLEBCOM cohort). Rectal swabs were processed in parallel by a culture-based and a qPCR-based method to quantitatively estimate the relative load of KPC-KP within the intestinal microbiota, based on the protocol originally described by Lerner *et al*. (reference 27). 18

Supplementary Figure S2. Correlation of the two methods used for relative quantification of intestinal KPC-KP load in rectal swabs. The graph shows the values of the culture-based method (% CFU of KPC-KP/ TAB) *versus* the qPCR-based method (% RL_{KPC}) for 80 rectal swab samples. 19

Supplementary Figure S3. Ranking of explanatory (independent) variables for 90-day mortality using the random survival forest score of importance. 20

Supplementary Figure S4. Distribution of RL_{KPC} values in hospitalized v*ersus* non-hospitalized patients. 21

Supplementary Figure S5. Distribution of RL_{KPC} values in the indicated groups of patients. P-values represent comparison between each patient group *versus* non-infected patients. The two episodes of surgical infections are not represented 22

Supplementary Figure S6. Receiver operator characteristic (ROC) curves (A) and optimum cut-off point, accuracy, sensitivity, and specificity for each infection group (B). 23

Supplementary Figure S7. Time-of-follow-up until infection or death without an infection at 90-days **(A)** and cumulative incidence function of competing events **(B)** for 80 patients with intestinal colonization by KPC-producing *Klebsiella pneumoniae*. 24

Supplementary Figure S8. Graphs for follow-up time until death or censoring (A), and survival function (B), for 80 patients with rectal colonization by KPC-producing *Klebsiella pneumoniae.* 25

Supplementary Figure S9. Optimal cut-off points for the variable "Length of hospital stay" (LOHS, days) with regards to mortality at 90 days (A) and 30 days (B). 26

Supplementary Figure S10. Schoenfeld residual tests for the final Cox regression models presented in **Table 3** after stratification by the variable "length of hospital stay". (**A**) Model for 90-day all-cause mortality in the global cohort. **(B)** Model for 30-day all-cause mortality in the global cohort (N=80). **(C)** Model for 90-day all-cause mortality in the subcohort of hospitalized patients. **(D)** Model for 30-day all-cause mortality in the subcohort of hospitalized patients. 27

Supplementary Methods

Culture-based and qPCR-based quantification of the relative load of KPC-KP in rectal swabs

Rectal swabs were processed within 4 hours of collection. The swab was vortexed for 1 minute to facilitate dilution of bacterial cell into the Amies Transport media. A 50 µL aliquot of this transport medium was submitted to serial dilutions and inoculation onto two types of selective plates for quantification of carbapenem-resistant *Enterobacterales* (CRE, CHROMID® CARBA plate, Biomeriéux, Marcy-l'Étoile, France), and total aerobic bacteria (TAB, Columbia agar plate supplemented with 5% sheep blood, Biomeriéux, Marcy-l'Étoile, France) following standard microbiological procedures. The (KPC-KP/TAB) colony forming unit (CFU) ratio was calculated and expressed as a percentage [1]. The remaining transport medium (approximately 900 µL) was centrifuged (10,000 x *g*, 10 minutes) and the pellet was stored at -80°C, until it was submitted to genomic DNA (gDNA) extraction with *UltraClean® Microbial DNA Isolation kit* (Qiagen, Hilden, Germany), according to the manufacturer´s instructions. qPCR was performed with iTaq™ Universal SYBR® Green Supermix (Bio-Rad, California, USA) in two singleplex assays, using 4 µl gDNA in a 20 µl final volume. The first singleplex assay was designed to estimate total bacteria based on analysis of the number of copies of the 16S rRNA gene with primers 515F (5'-GTGCCAGCAGCCGCGGTAA-3') and 685R (5'-TCTACGCATTTCACCGCTAC-3'). The second assay estimated the presence of KPC producers based on analysis of the copy number of *bla*$_{KPC}$ gene, with primers 15F (5'-CCGTCTAGTTCTGCTGTCTTGT-3') and 177R (5'-GTAACTTACAGTTGCGCCTGAG-3') (Nikkari et al. 2002; KeinÃ¤nen-Toivola, Revetta, and Santo Domingo 2006; Lerner et al.

2013a). These plasmids were also used to build standard curves for estimation of bla_{KPC} and 16SrRNA gene copy number in patient rectal swab samples. Cycling conditions were: 95°C for 5 min, 40 cycles of (5 sec at 95°C and 30 sec at 60°C), and a final melt curve analysis (65-95°C, 5°C increments at 5 sec/step). All reactions were carried out in a T100™ Thermal Cycler (Bio-Rad, California, USA), in duplicate. The threshold cycles (Ct) for bla_{KPC} and 16SrRNA genes were automatically calculated by the PCR system. Specificity of the qPCR products was manually inspected by melting curve analysis performed in the T100™ Thermal Cycler. The relative load of bla_{KPC} genes (RL_{KPC}) was defined as a percentage of the ratio: bla_{KPC} gene copies/ 16S rRNA gene copies.

Linearity, precision, and reportable ranges of the two independent qPCR reactions were established using serial dilutions of commercial cloning plasmids genetically engineered in our laboratory to harbor the 16S rRNA and bla_{KPC} gene fragments of interest. Efficiency (Ef) was calculated based on the standard curve slope ($Ef = 10^{(-1/slope)}-1$). The linear range of the bla_{KPC} qPCR was maintained for seven logarithmic dilutions (R^2 = 0.993, Ef=0.92), and the linear range of the 16S rRNA qPCR for seven logarithmic dilutions (R^2=0.993, Ef=0.91) (**Supplementary Table S2**). The limit of detection was set below 10^{-5} ng/µL KPC-KP gDNA for both 16SrRNA and bla_{KPC} qPCR assays.

Supplementary Tables

Supplementary Table S1. Checklist of items according to STROBE document.

	**Recommendation**	**Assessment in article**
Title and abstract	(*a*) Indicate the study's design with a commonly used term in the title or the abstract	Study design specified in the abstract
	(*b*) Provide in the abstract an informative and balanced summary of what was done and what was found	Balanced summary included in the abstract
Background/rationale	Explain the scientific background and rationale for the investigation being reported	The scientific background and rationale are included in the Importance Section and Introduction
Objectives	State specific objectives, including any prespecified hypotheses	Pre-specified hypotheses and objectives are stated in the Importance Section and Introduction
Study design	Present key elements of study design early in the paper	Study design described in the first part of Methods (Setting, patient selection and collection of rectal swabs)
Setting	Describe the setting, locations, and relevant dates, including periods of recruitment, exposure, follow-up, and data collection	Described in Methods
Participants	(*a*) Give the eligibility criteria, and the sources and methods of selection of participants. Describe methods of follow-up	Described in Methods
	(*b*) For matched studies, give matching criteria and number of exposed and unexposed	This is not a matched study
Variables	Clearly define all outcomes, exposures, predictors, potential confounders, and effect modifiers. Give diagnostic criteria, if applicable	Defined in Methods
Data sources/ measurement	For each variable of interest, give sources of data and details of methods of assessment (measurement). Describe comparability of assessment methods if there is more than one group	Specified in Methods
Bias	Describe any efforts to address potential sources of bias	Selection bias: inclusion of consecutive cases. Information bias: use of standard, well defined, easy to collect variables
Study size	Explain how the study size was arrived at	The sample size was calculated considering the retrospective data obtained from a previous cohort in our hospital. We calculated survival function and assumed 35% mortality in colonized patients, a statistic power of 80%, and a percentage of losses to follow-up of 5%. Based on this, a sample size of 57 colonized patients was estimated
Quantitative variables	Explain how quantitative variables were handled in the analyses. If applicable, describe which groupings were chosen and why	Quantitative variables were handled as such or dichotomized based on receiver operator curve

		(ROC) analysis for the outcome of interest
Statistical methods	(*a*) Describe all statistical methods, including those used to control for confounding	Included in Methods
	(*b*) Describe any methods used to examine subgroups and interactions	Included in Results
	(*c*) Explain how missing data were addressed	Patients with missing data were excluded from the main cohort for this analysis
	(*d*) If applicable, explain how loss to follow-up was addressed	Clinical data and clinical outcomes were obtained from hospital databases with the expressed informed consent of patients
	(*e*) Describe any sensitivity analyses	Included in Results
Participants	(a) Report numbers of individuals at each stage of study—e.g. numbers potentially eligible, examined for eligibility, confirmed eligible, included in the study, completing follow-up, and analyzed	Not recorded
	(b) Give reasons for non-participation at each stage	Not recorded
	(c) Consider use of a flow diagram	Not applicable
Descriptive data	(a) Give characteristics of study participants (e.g. demographic, clinical, social) and information on exposures and potential confounders	Table 1
	(b) Indicate number of participants with missing data for each variable of interest	The database was curated, and all variables of interest were obtained from hospital clinical records
	(c) *Cohort study*—Summarize follow-up time (e.g., average and total amount)	Included in Supplementary Figure S7
Outcome data	Cohort study—Report numbers of outcome events or summary measures over time	Table 1 and Supplementary Table S4
Main results	(*a*) Give unadjusted estimates and, if applicable, confounder-adjusted estimates and their precision (e.g., 95% confidence interval). Make clear which confounders were adjusted for and why they were included	Specified in Results
	(*b*) Report category boundaries when continuous variables were categorized	See Supplementary Figure S9 for categorization of LOHS variable
	(*c*) If relevant, consider translating estimates of relative risk into absolute risk for a meaningful time period	Not applicable
Other analyses	Report other analyses done—eg analyses of subgroups and interactions, and sensitivity analyses	Specified in Methods and Results
Key results	Summarize key results with reference to study objectives	Specified in Abstract, Important Section and Discussion
Limitations	Discuss limitations of the study, taking into account sources of potential bias or imprecision. Discuss both direction and magnitude of any potential bias	Included in Discussion
Interpretation	Give a cautious overall interpretation of results considering objectives, limitations, multiplicity of analyses, results from similar studies, and other relevant evidence	Included in Discussion
Generalizability	Discuss the generalizability (external validity) of the study results	Included in Discussion

Funding	Give the source of funding and the role of the funders for the present study and, if applicable, for the original study on which the present article is based	Included in Acknowledgements

Supplementary Table S2. Linearity of qPCR assays.

qPCR assay	Serial dilution sample	SYBR ™ Green SinglePlex qPCR		
		**Range (Log_{10} CFU)**	**Slope**	**R^2**
KPC-producing *K. pneumoniae*	*bla*$_{KPC3}$:pCR-Blunt II TOPO	2-9	-3.459	>0.99
	16S rRNA:pDrive	2-9	-3.378	>0.99

Supplementary Table S3. Antimicrobial susceptibility profiles of 80 KPC-producing *Klebsiella pneumoniae* isolates.

**Antimicrobial agent**	**S ≤ (mg/L)**	**R > (mg/L)**	**S (%)**	**I (%)**	**R (%)**	**MIC_{50} (mg/L)**	**MIC_{90} (mg/L)**
Ceftazidime/avibactam	8	8	100	0	0	>2	8
Colistin	2	2	89.0	0	11.0	0.5	>2
Tigecycline	1	2	35.4	59.7	4.9	2	2
Gentamicin	2	4	26.8	63.4	9.8	4	>4
Amikacin	8	16	3.7	1.2	95.1	>32	>32
Tobramycin	2	4	1.2	0	98.8	>8	>8
Fosfomycin	32	32	1.2	0	98.8	>512	>512
Meropenem	2	8	0	2.4	97.6	>16	>16
Imipenem	2	8	1.2	2.4	96.4	>8	>8
Ertapenem	0.5	1	0	0	100	>4	>4

S, Susceptible, standard dosing regimen; I, Susceptible, increased exposure; R, Resistant; MIC, Minimum inhibitory concentration.

Supplementary Table S4. Clinical characteristics and outcomes for 33 infection episodes.

Patient	Age	Sex	Hospitalization (day 0)	1st positive RS[a]	GRS	RL_{KPC} (%)	Time to infection from day 0 (days)	Time to infection from first detection of colonization (days)	Type of infection	ICS	Infection source	Antibiotic treatment	Time to death
**K081**	65	Male	Yes	1	7	9.30	6	7	BSI	15	Vascular access	CAZ/AVI	8
**K058**	71	Male	Yes	1	13	8.78	13	14	BSI	5	Biliary tract	CAZ/AVI	55
**K105**	88	Male	Yes	0	8	2.22	7	7	BSI	8	Urinary tract	CAZ/AVI	16
**K095**	73	Male	Yes	0	5	1.90	4	4	BSI	3	Urinary tract	CAZ/AVI	64
**K085**	85	Female	Yes	0	8	0.31	3	3	BSI	8	Urinary tract	CAZ/AVI	
**K086**	61	Female	Yes	0	8	0.29	2	2	BSI	3	Urinary tract	CAZ/AVI	42
**K015**	72	Male	Yes	0	8	0.02	15	15	BSI	13	Urinary tract and intrabdominal	CAZ/AVI	44
**K098**	84	Male	Yes	0	5	0.003	6	6	BSI	5	Urinary tract	CAZ/AVI	54
**K004**	70	Male	Yes	10	15	2.72	2	12	BSI & PNE	7	Vascular access and pneumonia	CAZ/AVI	83
**K030**	87	Female	Yes	10	5	7.78	3	13	UTI	5	Urinary tract	Fosfomycin	26
**K115**	84	Female	Yes	0	5	4.74	0	0	UTI	3	Urinary tract	CAZ/AVI	
**K104**	77	Female	Yes	2	5	4.74	5	7	UTI	3	Urinary tract	Fosfomicin	
**K103**	75	Male	Yes	0	5	1.01	3	3	UTI	5	Urinary tract	Gentamicin	
**K116**	91	Female	Yes	0	5	0.28	4	4	UTI	3	Urinary tract	CAZ/AVI	34
**K119**	81	Female	Yes	0	5	0.02	3	3	UTI	5	Urinary tract	CAZ/AVI	80
**K080**	88	Male	Yes	7	5	0.02	89	96	UTI	3	Urinary tract	CAZ/AVI	
**K100**	51	Male	Yes	2	8	0.01	4	6	UTI	3	Urinary tract	Cotrimoxazole	
**K087**	86	Female	No	0	5	0.01	25	25	UTI	0	Urinary tract	CAZ/AVI	
**K122**	88	Female	Yes	0	5	<0.001	5	5	UTI	3	Urinary tract	CAZ/AVI	
**K041**	83	Male	No	20	5	<0.001	27	47	UTI	5	Urinary tract	Cefiderocol	21
**K092**	89	Female	Yes	0	9	<0.001	0	0	UTI	3	Urinary tract	CAZ/AVI	
**K106**	84	Male	Yes	0	8	<0.001	2	2	UTI	3	Urinary tract	CAZ/AVI	
**K082**	50	Female	Yes	3	10	21.35	3	6	IAI	3	Intrabdominal	CAZ/AVI	
**K112**	55	Female	Yes	0	14	11.52	2	2	IAI	6	Intrabdominal	CAZ/AVI	
**K107**	76	Male	Yes	0	11	4.85	2	2	IAI	6	Intrabdominal	CAZ/AVI	
**K048**	85	Female	No	69	5	0.08	5	74	IAI	8	Intrabdominal	Gentamicin	

**K005**	85	Male	Yes	27	13	20.57	10	37	PNE	8	Pneumonia	Tigecycline	6
**K093**	44	Male	Yes	6	5	0.22	1	7	PNE	3	Pneumonia	CAZ/AVI	
**K006**	51	Female	Yes	3	7	0.12	3	6	PNE	3	Pneumonia	CAZ/AVI	
**K077**	81	Male	Yes	1	8	<0.001	2	3	PNE	6	Pneumonia	CAZ/AVI	5
**K099**	89	Male	Yes	0	5	<0.001	4	4	PNE	6	Pneumonia	CAZ/AVI	1
**K061**	71	Female	Yes	13	13	1.09	13	26	SSI	8	Skin and soft tissue	CAZ/AVI	29
**K114**	80	Female	Yes	0	8	0.36	6	6	SSI	8	Skin and soft tissue	CAZ/AVI	

BSI, bloodstream infection; CAZ/AVI, ceftazidime/avibactam; GRS, Giannella risk score; IAI, intrabdominal infection; ICS, INCREMENT-CPE Score; PNE, pneumonia; RL_{KPC}, relative load of KPC-KP within the gut microbiota; RS, rectal swab; SSI, surgical site infection; UTI, urinary tract infection.

[a] Days from recruitment (day 0 of follow-up) to first positive rectal swab, according to data extracted from hospital information systems. A negative value indicates diagnosis of KPC-KP colonization status in the previous 3 months.

Supplementary Table S5. Univariable competing risk Fine-Gray regression model for the first KPC-KP infection episode *versus* death from other causes (considered as competing risk) within 90 and 30 days of follow-up in the global cohort.

	90-day infection		30-day infection	
	Univariable analysis		Univariable analysis	
	SHR (95% CI)	p-value	SHR (95% CI)	p-value
Age (years), median (IQR)	**0.97 (0.95-0.99)**	**0.022**	**0.97 (0.94-0.99)**	**0.016**
Sex (Female)	1.50 (0.77-2.89)	0.230	1.40 (0.72-2.73)	0.320
Hospitalization				
Hospitalization in the previous 3 months	0.61 (0.48-7.08)	0.370	0.69 (0.19-20.93)	0.506
Hospitalization at recruitment	2.20 (0.75-6.46)	0.150	2.13 (0.73-6.16)	0.160
Intensive care unit admission	**3.05 (1.53-6.07)**	**0.002**	**3.09 (1.57-6.09)**	**0.001**
LOHS, median (IQR)	**1.01 (1.00-1.03)**	**0.039**	1.01 (0.10-1.03)	0.091
**Comorbidities**				
Charlson's score, median (IQR)	1.06 (0.89-1.25)	0.520	1.02 (0.85-1.22)	0.830
Diabetes mellitus	0.81 (0.41-1.59)	0.530	0.75 (0.37-1.50)	0.410
Chronic renal disease	1.45 (0.71-2.96)	0.300	1.51 (0.74-3.06)	0.260
Tumor	0.21 (0.58-2.55)	0.610	1.03 (0.46-2.34)	0.930
**McCabe's score**				
Non-fatal	0.85 (0.34-2.10)	0.720	0.89 (0.36-2.18)	0.790
Rapidly fatal	0.73 (0.365-1.50)	0.400	0.77 (0.38-1.58)	0.480
Ultimately fatal	1.49 (0.77-2.86)	0.240	1.38 (0.71-2.69)	0.340
**Clinical factors prior to recruitment**				
Immunosuppressive therapy	1.46 (0.67-3.18)	0.340	1.51 (0.70-3.26)	0.290
Recurrent UTI	0.76 (0.27-2.13)	0.600	0.78 (0.28-2.20)	0.640
Antibiotic exposure in the previous month	1.4 (0.28-6.89)	0.680	1.34 (0.27-6.58)	0.720
Amoxicillin/Clavulanic Acid	0.83 (0.37-1.87)	0.660	0.87 (0.39-1.94)	0.730
Piperacillin/Tazobactam	1.29 (0.65-2.58)	0.470	1.17 (0.57-2.41)	0.670
Cephalosporins	1.24 (0.63-2.42)	0.530	1.30 (0.66-2.56)	0.440
Carbapenems	0.86 (0.28-2.68)	0.800	0.89 (0.29-2.75)	0.840
Aminoglycosides	0.93 (0.34-2.49)	0.880	0.96 (0.36-2.56)	0.930
Fluoroquinolones	1.70 (0.88-3.28)	0.120	1.80 (0.92-3.51)	0.087
Invasive procedures in the previous month	1.54 (0.58-4.12)	0.390	1.48 (0.56-3.93)	0.430
Urinary catheter	1.68 (0.72-3.91)	0.230	1.61 (0.69-3.73)	0.270
Nasogastric intubation	0.95 (0.41-2.17)	0.900	0.99 (0.43-2.25)	0.970
Surgical procedures	**2.34 (1.17-4.70)**	**0.020**	**2.42 (1.21-4.84)**	**0.012**
Central venous catheter	**1.98 (0.99-3.94)**	**0.050**	**2.03 (1.02-4.05)**	**0.043**
Mechanical ventilation	1.96 (0.91-4.20)	0.090	2.01 (0.94-4.29)	0.071
Endoscopic procedures	2.02 (0.79-5.17)	0.140	2.07 (0.82-5.22)	0.120
**Clinical factors during follow-up**				
Giannella risk score, median (IQR)	**1.24 (1.17-1.32)**	**<0.001**	**1.24 (1.17-1.33)**	**<0.001**
Central venous catheter (first month)	**2.91 (1.38-6.13)**	**0.005**	**2.97 (1.42-6.21)**	**0.004**
**RL_{KPC} (%) on day 0, median (IQR)**	0.99 (0.96-1.03)	0.680	0.99 (0.96-1.03)	0.740

CI, confidence interval, IQR, interquartile range; LOHS, length of hospital stay; RL_{KPC}, relative load of KPC-KP within the gut microbiota; SHR, sub-distribution hazard ratio; UTI, urinary tract infection.

Supplementary Table S6. Univariable and multivariable Cox regression analyses of risk factors for 90-day mortality in the global cohort of 80 patients.

	Dead	Alive	Univariable analysis		Multivariable analysis	
	N=33	N=47	HR (95% CI)	p-value	Adjusted HR (95% CI)	p-value
Age (years), median (IQR)	**85 (81-89)**	**83 (68-87)**	**1.04 (1.00-1.08)**	**0.033**	**1.05 (1.01-1.09)**	**0.010**
Sex (Male)	16 (48.5)	18 (38.3)	1.35 (0.68-2.68)	0.384		
Hospitalization						
Hospitalization in the previous 3 months	12 (36.4)	14 (29.8)	1.24 (0.63-2.47)	0.535		
Hospitalization at recruitment	31 (93.9)	37 (78.7)	3.14 (0.75-13.14)	0.117		
Intensive care unit admission	4 (12.9)	3 (8.1)	1.38 (0.48-3.92)	0.548		
LOHS, median (IQR)	23 (16-39)	13 (7-24)	1.01 (0.10-1.02)	0.193		
LOHS ≥ 20 days, median (IQR)	**23 (69.7)**	**16 (34.0)**	**2.73 (1.30-53.75)**	**0.008**	**2.85 (1.32-6.18)** [d]	**0.008**
**Comorbidities**						
Charlson's score, median (IQR)	3 (2-4)	2 (1-3)	1.18 (0.98-1.41)	0.078		
Diabetes mellitus	14 (42.4)	21 (44.7)	0.88 (0.44-1.75)	0.708		
Chronic renal disease	**11 (33.3)**	**6 (12.8)**	**2.28 (1.10-4.72)**	**0.026**		
Tumor	8 (24.2)	6 (12.8)	1.93 (0.87-4.29)	0.106		
McCabe score						
Non-fatal	**3 (9.1)**	**14 (29.8)**	**0.30 (0.09-0.98)**	**0.046**		
Rapidly fatal	9 (27.3)	23 (48.9)	0.52 (0.24-1.13)	0.098		
Ultimately fatal	**21 (63.6)**	**10 (21.3)**	**3.58 (1.75-7.31)**	**<0.001**		
**Clinical factors prior to recruitment**						
Immunosuppressive therapy	**9 (27.3)**	**4 (8.5)**	**2.39 (1.11-5.15)**	**0.026**		
Recurrent UTI	6 (18.2)	6 (12.8)	1.31 (0.54-3.16)	0.555		
Antibiotic exposure in the previous month	32 (97)	41 (87.2)	3.49 (0.48-25.62)	0.218		
Amoxicillin/Clavulanic Acid	6 (18.2)	13 (27.7)	0.70 (0.29-1.69)	0.427		
Piperacillin/Tazobactam	7 (21.2)	16 (34)	0.58 (0.25-1.33)	0.200		
Cephalosporins	13 (39.4)	18 (38.3)	1.00 (0.48-1.95)	0.929		
Carbapenems	5 (15.2)	3 (6.4)	1.81 (0.70-4.71)	0.221		
Aminoglycosides	6 (18.2)	4 (8.5)	1.78 (0.74-4.32)	0.200		
Fluoroquinolones	16 (48.5)	15 (31.9)	1.73 (0.87-3.42)	0.117		
Invasive procedures in the previous month	28 (84.8)	35 (74.5)	1.51 (0.58-3.92)	0.396		
Urinary catheter	25 (75.8)	31 (66.0)	1.25 (0.56-2.77)	0.585		

Nasogastric intubation	7 (21.2)	11 (23.4)	0.89 (0.39-2.05)	0.783		
Surgical procedures	4 (12.1)	11 (23.4)	0.51 (0.18-1.45)	0.209		
Mechanical ventilation	6 (18.2)	5 (10.6)	1.63 (0.67-3.96)	0.278		
Central venous catheter	7 (21.2)	7 (14.9)	1.23 (0.53-2.84)	0.625		
Endoscopic procedures	2 (6.1)	4 (8.5)	0.75 (0.18-3.15)	0.697		
**Clinical factors during follow-up**						
Central venous catheter (first month)	5 (15.2)	4 (8.5)	1.52 (0.59-3.95)	0.386		
Antibiotic exposure (first month)	31 (93.9)	37 (78.7)	3.22 (0.77-13.50)	0.109		
Amoxicillin/Clavulanic Acid	7 (21.2)	9 (19.1)	1.05 (0.46-2.43)	0.901		
Piperacillin/Tazobactam	14 (42.4)	16 (34)	1.42 (0.71-2.83)	0.321		
Ceftazidime/avibactam	13 (39.4)	12 (25.5)	1.32 (0.66-2.66)	0.432		
Cephalosporins	11 (33.3)	16 (34.0)	0.97 (0.47-2.00)	0.936		
carbapenems	4 (12.1)	2 (4.3)	1.80 (0.63-5.14)	0.270		
Aminoglycosides	**8 (24.2)**	**4 (8.5)**	**2.27 (1.02-5.05)**	**0.044**		
Fluoroquinolones	8 (24.2)	6 (12.8)	1.99 (0.90-4.42)	0.091		
**Infection episode**	16 (48.5)[c]	17 (36.2)	1.29 (0.65-2.56)	0.462		
Giannella risk score, median (IQR)	5 (5-8)	5 (5-8)	0.99 (0.90-1.10)	0.913		
Type of infection						
Bloodstream infection	**8 (24.2)**	**1 (2.1)**	**2.66 (1.19-5.92)**	**0.017**		
Urinary-tract infection	4 (12.1)	9 (19.1)	0.60 (0.21-1.72)	0.345		
Intrabdominal infection	0 (0)	4 (8.5)	--	0.997		
Pneumonia	4 (12.1)	2 (4.3)	2.40 (0.84-6.84)	0.101		
Surgical infection	1 (3.0)	1 (2.1)	1.21 (0.17-8.88)	0.850		
INCREMENT-CPE Score, median (IQR)[a]	**5.5 (5-8)**	**3 (3-6)**	**1.22 (1.06-1.41)**	**0.007**		
Severe KPC-KP infection[b]	5 (15.2)	3 (6.4)	2.00 (0.77-5.19)	0.155	1.88 (0.71-4.94)	0.204
**RL_{KPC} (%) on day 0, median (IQR)**	**0.30 (0.01-7.55)**	**0.22 (0.01-1.51)**	**1.03 (1.01-1.07)**	**0.006**	**1.03 (1.00-1.06)**	**0.027**

CI, confidence interval, IQR, interquartile range; LOHS, length of hospital stay; RL_{KPC}, relative load of KPC-KP within the gut microbiota; UTI, urinary tract infection.

Data are number (%) for categorical variables or median (IQR) for continuous variables.

[a] INCREMENT-CPE score, risk of mortality in patients who develop an infection by a carbapenemase-producing *Enterobacterales* (6). The median (IQR) was calculated only for 33 patients with KPC-KP infection episodes.

[b] Severe KPC-KP infection was considered in patients with a KPC-KP infection and an INCREMENT-CPE Score >7 (6, 7).

[c] One patient developed two infection episodes, i.e. a bloodstream infection and a pneumonia episode.

[d] The variable LOHS did not fulfill the proportional hazards assumption in this model, so it was used for stratification of the final multivariable model presented in **Tables 3A** and **3C**.

Supplementary Table S7. Univariable and multivariable Cox regression analyses of risk factors for 30-day mortality in the global cohort of 80 patients.

	Dead	Alive	Univariable analysis		Multivariable analysis	
	N=22	N=58	HR (95% CI)	p-value	Adjusted HR (95% CI)	p-value
Age (years), median (IQR)	**86 (82-89)**	**82 (71-87)**	**1.07 (1.01-1.13)**	**0.019**	**1.10 (1.03-1.17)**	**0.006**
Sex (Male)	10 (45.5)	24 (41.4)	1.20 (0.52-2.78)	0.670		
**Hospitalization**						
Hospitalization at recruitment	18 (81.8)	33 (56.9)	1.87 (0.44-8.02)	0.397		
Intensive care unit admission	2 (9.1)	5 (8.6)	0.99 (0.23-4.22)	0.990		
LOHS, median (IQR)	21 (15-26)	17 (8-35)	0.10 (0.98-1.02)	0.952		
LOHS ≥ 14 days, median (IQR)	18 (81.8)	33 (56.9)	2.68 (0.91-7.93)	0.075	**3.37 (1.11-10.27)[d]**	**0.032**
**Comorbidities**						
Charlson's score, median (IQR)	3 (2-4)	2 (1-3)	1.67 (0.93-1.46)	0.178		
Diabetes mellitus	9 (40.9)	26 (44.8)	0.84 (0.36-1.96)	0.683		
Chronic renal disease	**9 (40.9)**	**8 (13.8)**	**2.77 (1.18-6.50)**	**0.019**		
Tumor	5 (22.7)	9 (15.5)	1.67 (0.62-4.54)	0.311		
Mc Cabe's score						
Non-fatal	1 (4.5)	16 (27.6)	0.16 (0.02-1.15)	0.069		
Rapidly fatal	8 (36.4)	24 (41.4)	0.86 (0.36-2.05)	0.735		
Ultimately fatal	**13 (59.1)**	**18 (31.0)**	**2.50 (1.07-5.86)**	**0.034**		
**Clinical factors prior to recruitment**						
Immunosuppresive therapy	6 (27.3)	7 (12.1)	2.19 (0.86-5.61)	0.101		
Recurrent UTI	4 (18.2)	8 (13.8)	1.25 (0.42-3.71)	0.682		
Antibiotic exposure in the previous month	21 (95.5)	52 (89.7)	2.10 (0.28-15.61)	0.470		
Amoxicillin/Clavulanic Acid	6 (27.3)	13 (22.4)	1.20 (0.47-3.06)	0.710		
Piperacillin/Tazobactam	3 (13.6)	20 (34.5)	0.36 (0.11-1.23)	0103		
Cephalosporins	8 (36.4)	23 (39.7)	0.82 (0.34-1.94)	0.647		
Carbapenems	5 (8.6)	3 (13.6)	1.45 (0.43-4.91)	0.548		
Aminoglycosides	4 (18.2)	6 (10.3)	1.71 (0.58-5.07)	0.331		
Fluoroquinolones	10 (45.5)	23 (39.7)	1.36 (0.59-3.16)	0.467		
Invasive procedures in the previous month	18 (81.8)	45 (77.6)	1.10 (0.37-3.25)	0.865		
Urinary catheter	15 (68.2)	41 (70.7)	0.78 (0.32-1.93)	0.596		
Nasogastric intubation	3 (13.6)	15 (25.9)	0.53 (0.16-1.78)	0.302		
Surgical procedures	2 (9.1)	13 (22.4)	0.38 (0.09-1.64)	0.195		
Mechanical ventilation	5 (22.7)	6 (10.3)	2.09 (0.77-5.69)	0.145		

Central venous catheter	3 (13.6)	11 (19.0)	0.72 (0.21-2.43)	0.594		
Endoscopic procedures	1 (4.5)	5 (8.6)	0.57 (0.08-4.23)	0.581		
**Clinical factors during follow-up**						
Central venous catheter (first month)	3 (13.6)	6 (10.3)	1.31 (0.39-4.42)	0.665		
Antibiotic exposure (first month)	20 (90.9)	48 (82.8)	2.10 (0.28-15.61)	0.469		
Amoxicillin/Clavulanic Acid	6 (27.3)	10 (17.2)	1.20 (0.47-3.06)	0.709		
Piperacillin/Tazobactam	10 (45.5)	20 (34.5)	0.36 (0.11-1.23)	0.103		
Ceftazidime/avibactam	4 (18.2)	21 (36.2)	1.64 (0.22-12.19)	0.630		
Cephalosporins	8 (36.4)	19 (32.8)	0.82 (0.34-1.94)	0.646		
Carbapenems	2 (9.1)	4 (6.9)	1.45 (0.43-4.91)	0.458		
Aminoglycosides	5 (22.7)	7 (12.1)	1.71 (0.58-5.07)	0.331		
Fluoroquinolones	**7 (31.8)**	**7 (12.1)**[c]	**2.73 (1.11-6.70)**	**0.028**		
**Infection episode**	8 (36.4)	24 (41.4)	0.82 (0.34-1.96)	0.654		
Giannella risk score, median (IQR)	5 (0-7)	5 (5-8)	0.94 (0.83-1.07)	0.377		
Infection type						
Bloodstream infection	2 (9.1)	7 (12.1)	0.76 (0.18-3.26)	0.710		
Urinary tract infection	2 (9.1)	10 (17.2)	0.44 (0.10-1.90)	0.272		
Intrabdominal infection	0 (0)	4 (6.9)	--	0.997		
Pneumonia	3 (13.6)	3 (5.2)	2.82 (0.83-9.55)	0.096		
Surgical infection	1 (4.5)	1 (1.7)	1.64 (0.22-12.19)	0.629		
INCREMENT-CPE Score, median (IQR)[a]	**7 (5-8)**	**3 (3-6)**	**1.24 (1.04-1.48)**	**0.014**		
Severe KPC-KP infection[b]	4 (18.2)	4 (6.9)	2.23 (0.75-6.59)	0.148	2.63 (0.87-7.98)	0.088
**RL_{KPC} (%) on day 0, median (IQR)**	**0.21 (<0.001-8.16)**	**0.28 (0.01-2.35)**	**1.04 (1.01-1.07)**	**0.011**	**1.03 (1.00-1.06**	**0.034**

CI, confidence interval, IQR, interquartile range; LOHS, length of hospital stay; RL_{KPC}, relative load of KPC-KP within the gut microbiota; UTI, urinary tract infection.

Data are number (%) for categorical variables or median (IQR) for continuous variables.

[a] INCREMENT-CPE score, risk of mortality in patients who develop an infection by a carbapenemase-producing *Enterobacterales* (6). The median (IQR) was calculated only for 33 patients with KPC-KP infection episodes.

[b] Severe KPC-KP infection was considered in patients with infections and an INCREMENT-CPE Score >7 (6, 7).

[c] One patient developed two infection episodes, a bloodstream infection and a pneumonia episode.

[d] The variable LOHS did not fulfill the proportional hazards assumption in this model, so it was used for stratification of the final multivariable model presented in **Table 3B** and **3D**.

Supplementary Figures

Supplementary Figure S1. Graphical summary of rectal swab processing in this study (KLEBCOM cohort). Rectal swabs were processed in parallel by a culture-based and a qPCR-based method to quantitatively estimate the relative load of KPC-KP within the intestinal microbiota, based on the protocol originally described by Lerner *et al*. (reference 27).

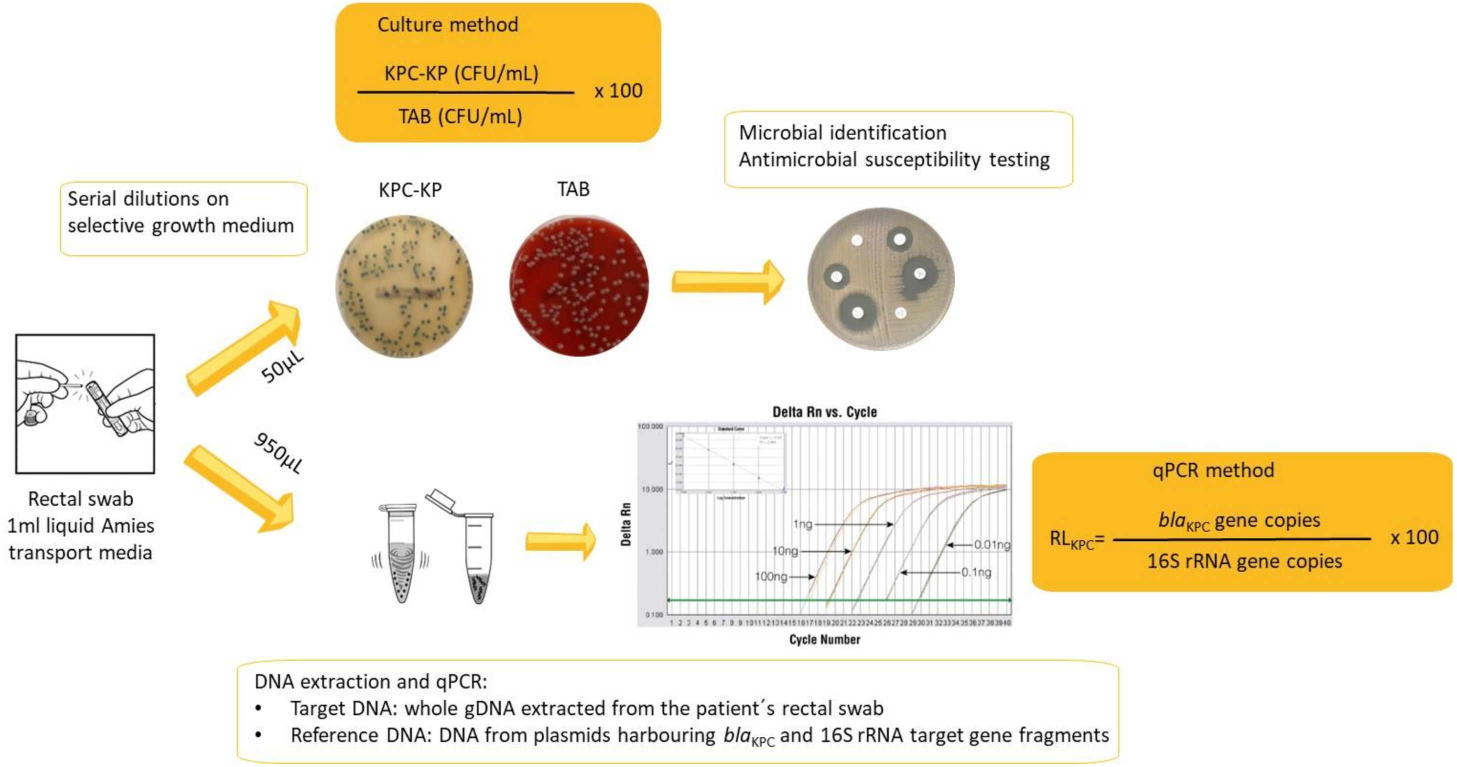

CFU, colony forming unit; RL_{KPC}, relative load of KPC-KP within the gut microbiota; Rn, normalized reporter; TAB, total aerobic bacteria.

Supplementary Figure S2. Correlation of the two methods used for relative quantification of intestinal KPC-KP load in rectal swabs. The graph shows the values of the culture-based method (% CFU of KPC-KP/ TAB) *versus* the qPCR-based method (% RL_{KPC}) for 80 rectal swab samples.

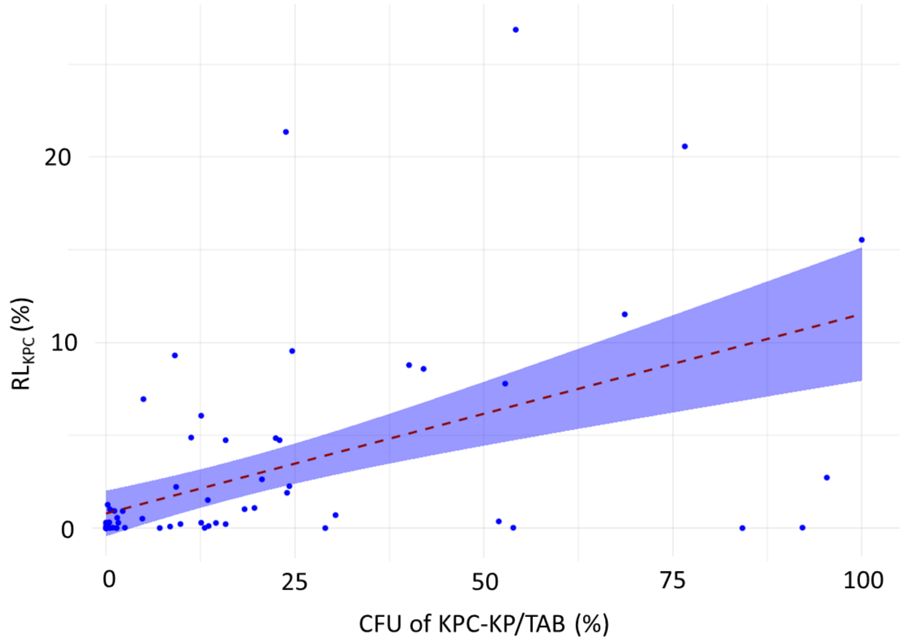

CFU, colony forming unit; RL_{KPC}, relative load of KPC-KP within the gut microbiota; TAB, total aerobic bacteria.

Kendall's rank correlation tau test= 0.494528, p-value = <0.001

Supplementary Figure S3. Ranking of explanatory (independent) variables for 90-day mortality using the random survival forest score of importance.

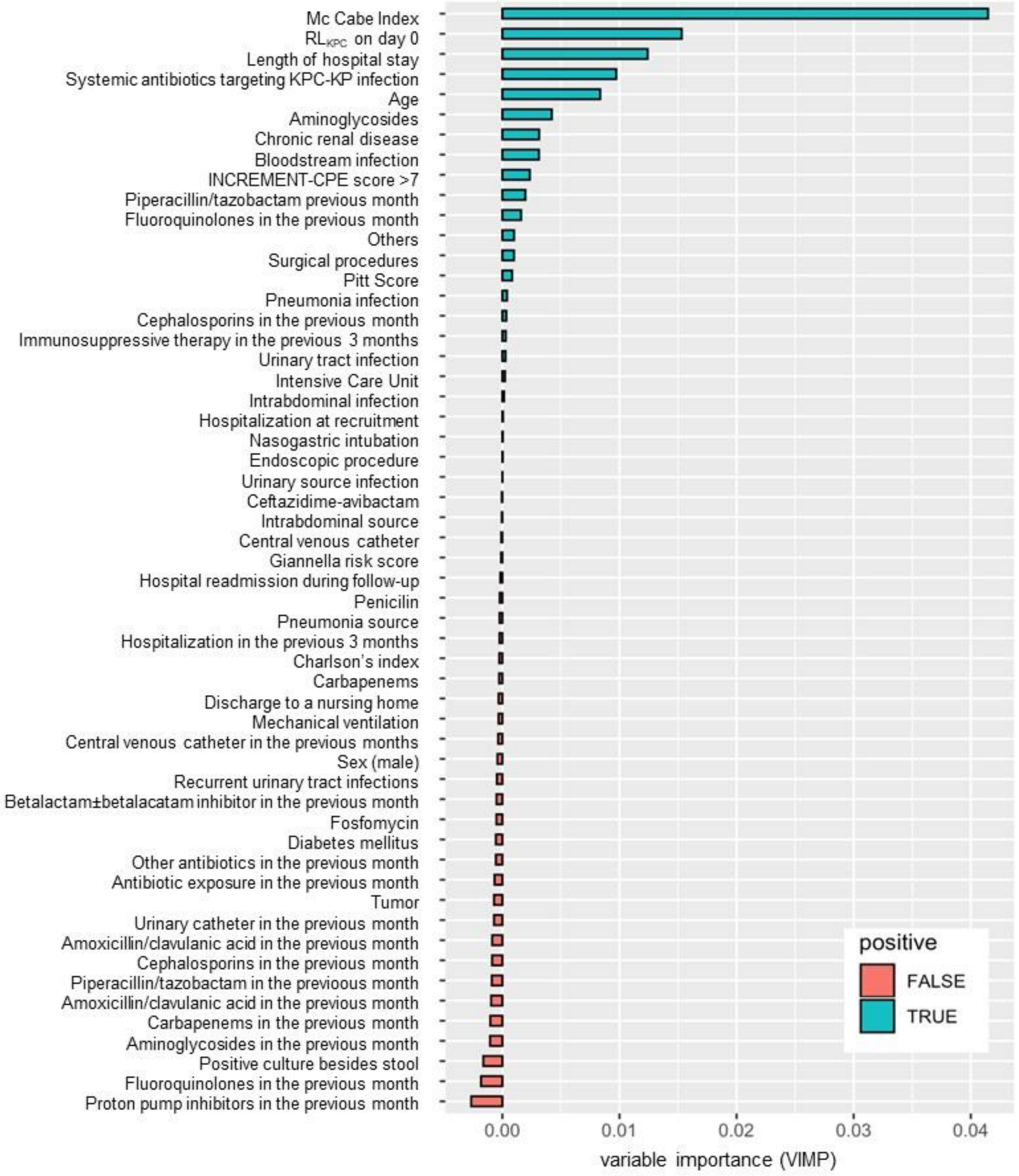

Supplementary Figure S4. Distribution of RL_{KPC} values in hospitalized v*ersus* non-hospitalized patients.

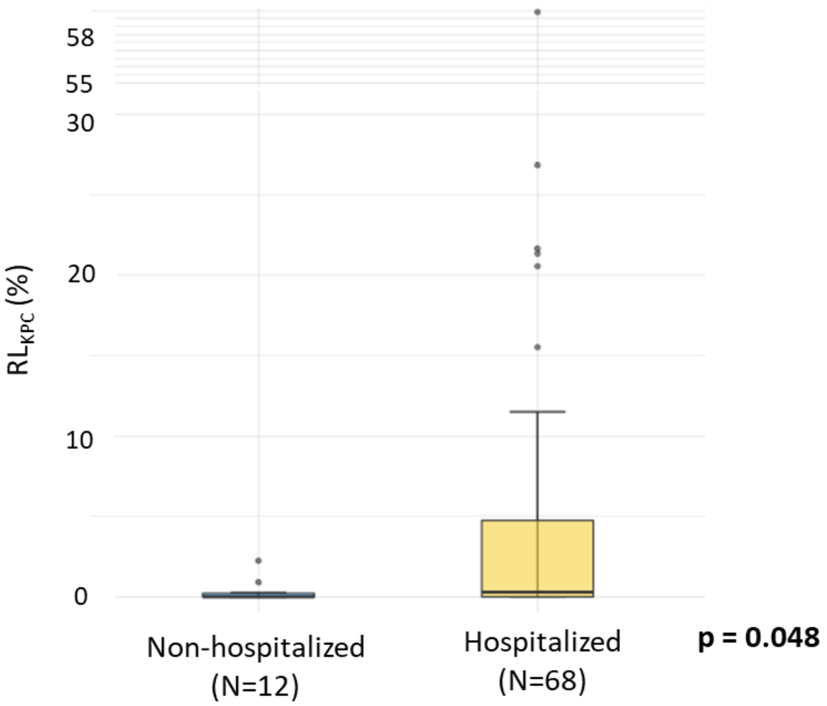

Supplementary Figure S5. Distribution of RL_{KPC} values in the indicated groups of patients. P-values represent comparison between each patient group *versus* non-infected patients. The two episodes of surgical infections are not represented.

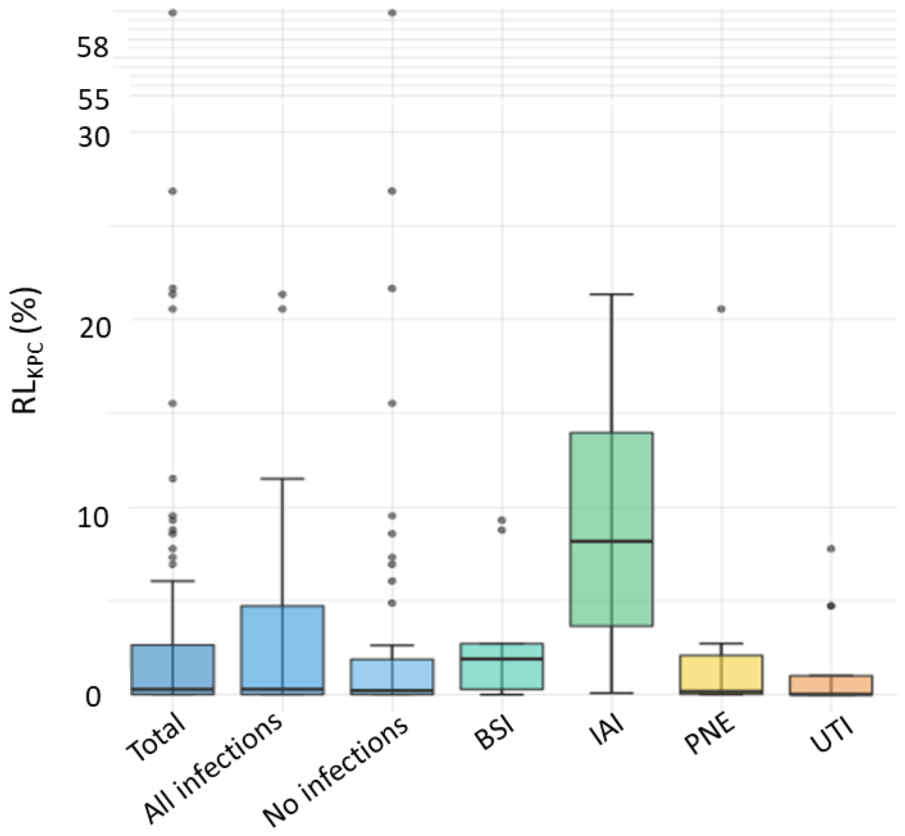

	Mann-Whitney (p-values)
No infections (N=47)	Ref.
All infections (N=33)	0.657
UTI (N=13)	0.286
BSI (N=9)	0.418
PNE (N=5)	0.810
IAI (N=4)	**0.017**
SSI (N=2)	0.463

BSI, Bloodstream infection; IAI, Intrabdominal infection; PNE, pneumonia; SSI, Surgical site infection; UTI, Urinary tract infection. RL_{KPC}, relative load of KPC-KP within the gut microbiota.

Supplementary Figure S6. Receiver operator characteristic (ROC) curves (A) and optimum cut-off point, accuracy, sensitivity, and specificity for each infection group (B).

A

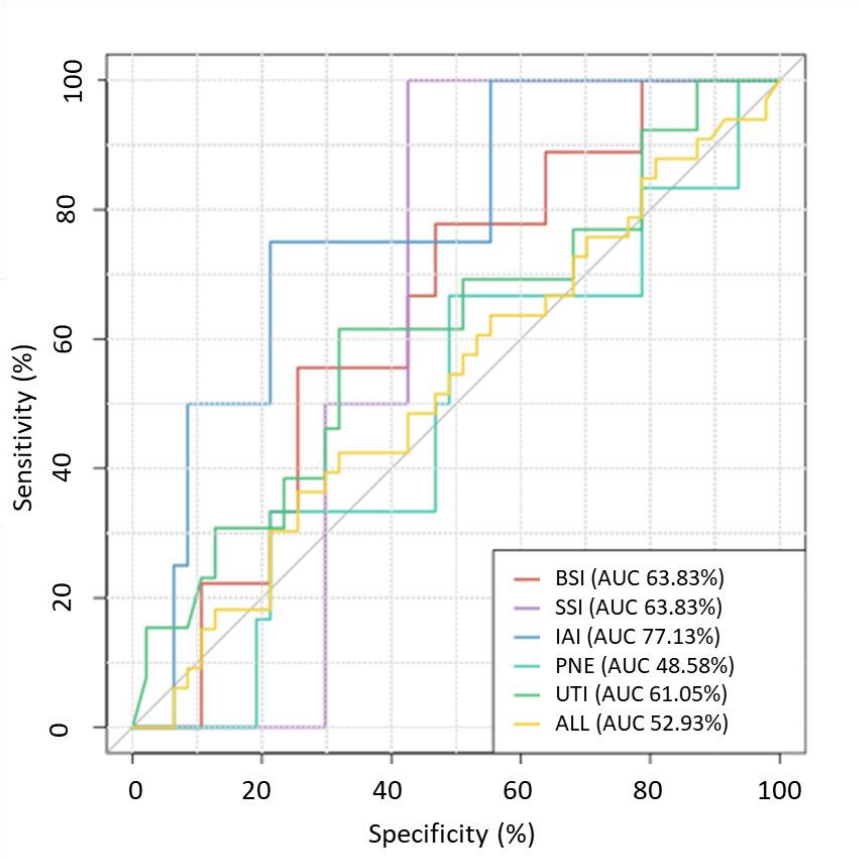

B

Infection type	Optimun cut-off	Accuracy (95% CI)	Sensitivity	Specificity
All-site	1.7	0.54 (0.42-0.65)	0.48	0.57
BSI	0.3	0.43 (0.30-0.57)	0.78	0.36
UTI	0.0	0.25 (0.15-0.38)	0.62	0.15
IAI	3.7	0.71 (0.56-0.83)	0.25	0.74
SSI	0.3	0.43 (0.29-0.58)	1.00	0.40
PNE	0.2	0.34 (0.22-0.48)	0.50	0.32

AUC, area under the curve; BSI, bloodstream infection; IAI, intrabdominal infection; PNE, pneumonia; SSI, Surgical site infection; UTI, urinary tract infection.

Supplementary Figure S7. Time-of-follow-up until infection or death without an infection at 90-days **(A)** and cumulative incidence function of competing events **(B)** for 80 patients with intestinal colonization by KPC-producing *Klebsiella pneumoniae*.

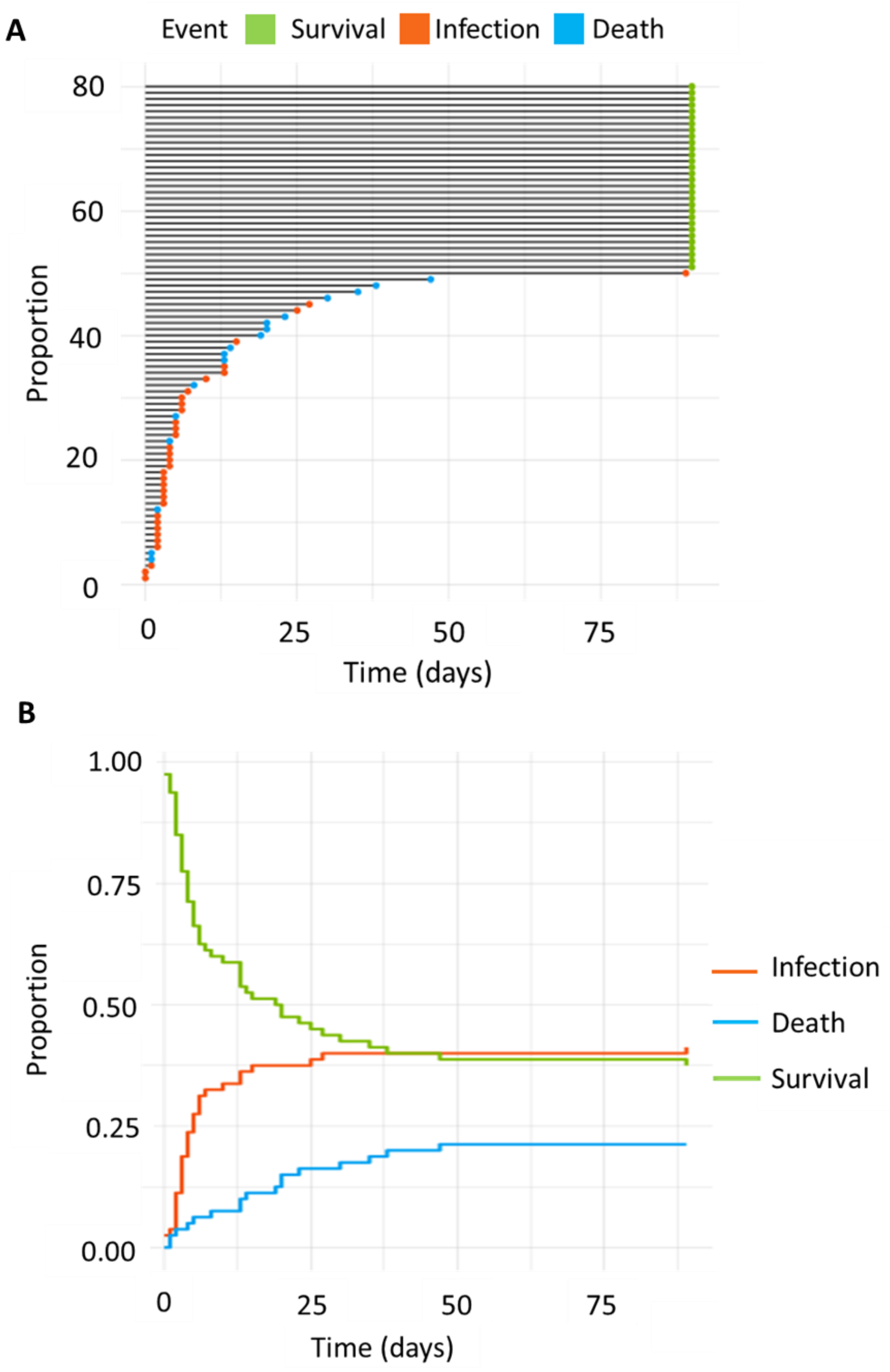

Supplementary Figure S8. Graphs for follow-up time until death or censoring (A), and survival function (B), for 80 patients with rectal colonization by KPC-producing *Klebsiella pneumoniae.*

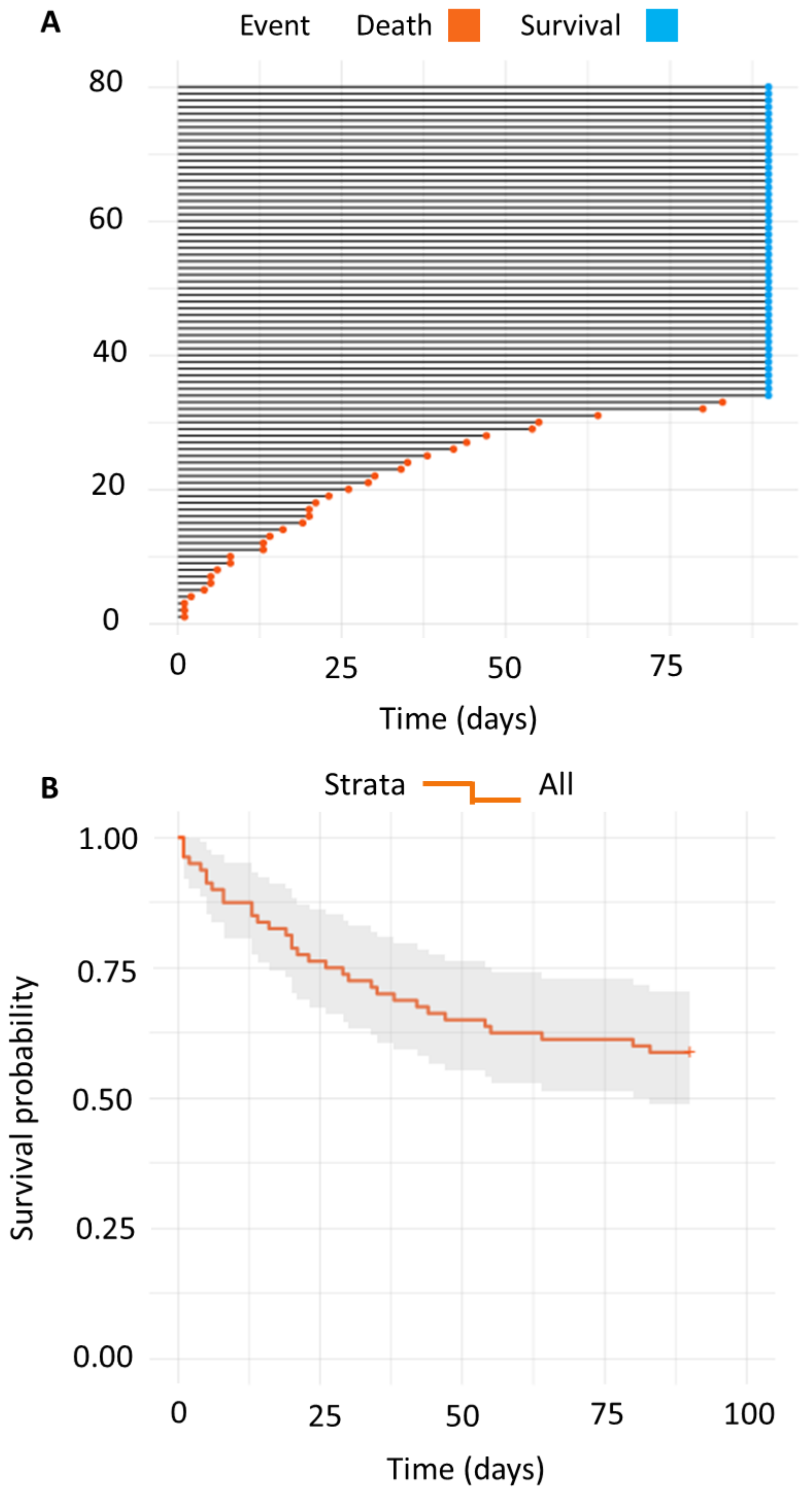

Supplementary Figure S9. Optimal cut-off points for the variable "Length of hospital stay" (LOHS, days) with regards to mortality at 90 days (A) and 30 days (B).

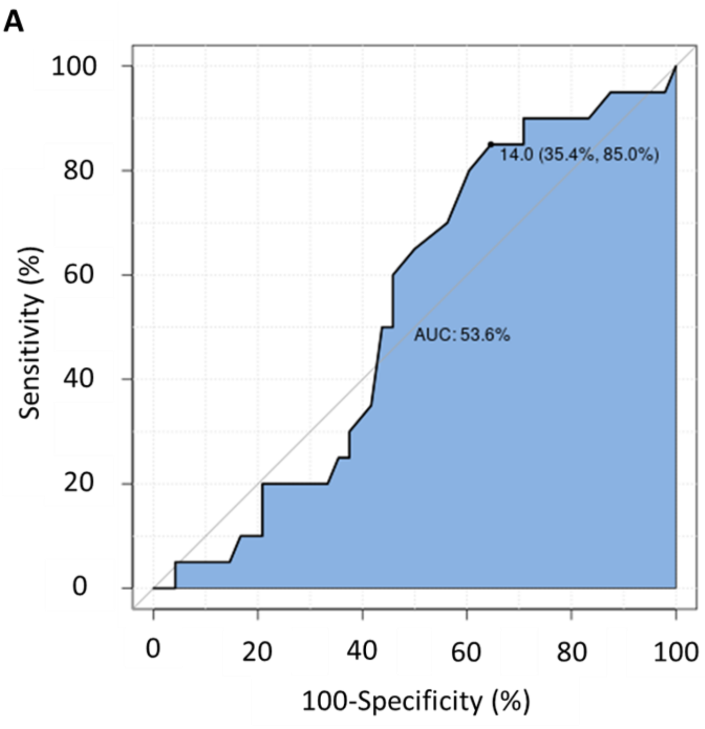

AUC, area under the curve.

Supplementary Figure S10. Schoenfeld residual tests for the final Cox regression models presented in **Table 3** after stratification by the variable "length of hospital stay". (**A**) Model for 90-day all-cause mortality in the global cohort. **(B)** Model for 30-day all-cause mortality in the global cohort (N=80). **(C)** Model for 90-day all-cause mortality in the subcohort of hospitalized patients. **(D)** Model for 30-day all-cause mortality in the subcohort of hospitalized patients.

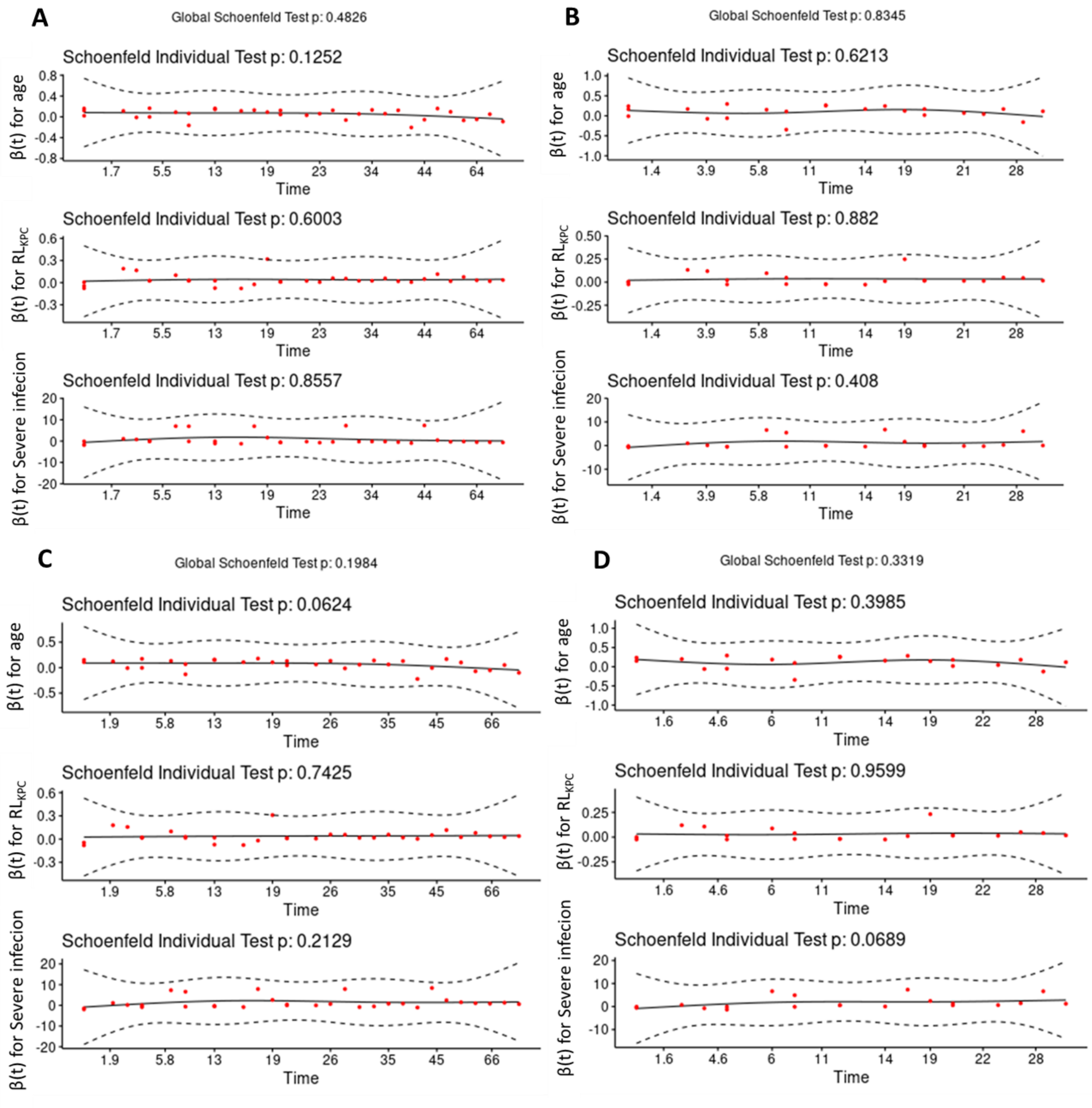

Supplement: Supplemental file 1 — Supplemental material. Download spectrum.02728-21-s0001.pdf, PDF file, 1.7 MB [file spectrum.02728-21-s0001.pdf]
